# Implantable Port Catheters versus Peripherally Inserted Central Catheters for Cancer Patients Requiring Chemotherapy: An RCT-Based Meta-Analysis

**DOI:** 10.7150/jca.103631

**Published:** 2025-01-06

**Authors:** Juan Qiu, Shanshan Huang, Pei Wen, Yingxin Jiang, Zan Luo, Wenxiong Zhang, Jianyun Wen

**Affiliations:** 1 Department of Thoracic Oncology Radiation Therapy, Jiangxi Cancer Hospital & Institute, Nanchang, China, 330029.; 2 Department of Thoracic Surgery, The Second Affiliated Hospital, Jiangxi Medical College, Nanchang University, Nanchang, China, 330006.; 3 Department of Head and Neck Oncology Radiation Therapy, Jiangxi Cancer Hospital & Institute, Nanchang, China, 330029.

**Keywords:** Implantable port catheters, Peripherally inserted central catheter, Complication, Meta-analysis, Randomized controlled trials

## Abstract

**Background:** Implantable port catheters (IPCs) and peripherally inserted central catheters (PICCs) are commonly used venous access methods for chemotherapy in cancer patients. However, the question of which is superior remains controversial. This meta-analysis, based on randomized controlled trials (RCTs), systematically compares the safety, cost, and impact on quality of life between these two methods.

**Methods:** Eligible RCTs comparing IPC and PICC were identified through searches in seven databases. Complications were the primary endpoint, while secondary endpoints included cost, impact on chemotherapy, and quality of life assessments.

**Results:** Six studies based on five RCTs, including a total of 1,127 patients, were analyzed. Patient data indicated that the PICC group experienced a higher incidence of total complications, thrombosis, deep vein thrombosis, implantation failure, unplanned catheter removal, and local reactions. Conversely, the IPC group had a higher incidence of pocket infection/exit-site infection without septicemia and pain. When considering catheter days, the PICC group again showed a higher incidence of total complications, thrombosis, deep vein thrombosis, implantation failure, unplanned catheter removal, edema, and local reactions. Complication-free survival was better in the IPC group. Although the impact on chemotherapy tended to favor the IPC group, this difference was not statistically significant. The total cost was higher in the IPC group, while the cost per catheter day was similar between the two groups. Quality of life assessments (using EORTC QLQ-C30) revealed similar global health status between the two groups during the post-implantation, mid-treatment, and end-treatment periods. However, the IPC group experienced a smaller decline in global health status post-implantation compared to the PICC group.

**Conclusions:** Compared to PICC, IPC appears to be a safer and more comfortable intravenous catheterization option for cancer patients undergoing chemotherapy.

## Introduction

Reliable venous access is essential for cancer patients undergoing chemotherapy, with implantable port catheters (IPCs) and peripherally inserted central catheters (PICCs) being the most commonly used options. The choice between these two devices remains a topic of clinical debate due to differences in safety profiles, patient comfort, and complication risks 1,2. IPCs, surgically implanted under the skin, provide direct access to central veins and are associated with lower rates of catheter-related infections (CRIs) due to less frequent access and a reduced risk of bacterial colonization 3. Conversely, PICCs, which are inserted through a peripheral vein, are easier to place and remove, making them preferable for patients who require rapid access or have contraindications to surgery 4.

However, PICCs are more prone to infections and venous thromboembolism (VTE), particularly in patients receiving thrombogenic chemotherapy or those with pre-existing hypercoagulable conditions 5. Studies, including those by Bertoglio *et al.* and Benvenuti *et al.*, highlight the higher risk of VTE associated with PICCs compared to IPCs, which generally have lower thrombosis rates 4,6. Despite the increased risks of infection and thrombosis, PICCs are often preferred in settings where lower initial costs and procedural simplicity are prioritized 7.

Patient comfort is another critical factor. IPCs, being fully implanted, are less intrusive and require less frequent maintenance, contributing to higher patient satisfaction and better adherence to treatment 8,9. Conversely, PICCs, which are visible and require regular care, may lead to increased anxiety and discomfort, potentially impacting the patient's quality of life 10-12.

Previous meta-analyses have often included retrospective studies, which are limited by inherent biases that compromise the reliability of their conclusions. By focusing exclusively on randomized controlled trials (RCTs), our study ensures the highest level of evidence for evaluating the safety, efficacy, and quality of life impacts of these venous access devices.

## Materials and methods

### Search strategy

RCTs comparing IPC and PICC were systematically searched in the Web of Science, EMBASE, Cochrane Library, PubMed, ScienceDirect, PMC (PubMed Central), and Scopus databases up to December 3, 2024. The MeSH terms used included 'Implantable port catheter' and 'Peripherally inserted central venous catheter'. Additionally, eligible articles were further identified by reviewing references from the retrieved literature. Detailed retrieval strategies are provided in **Table S1**.

### Selection criteria

Inclusion criteria:

(1) Population: Cancer patients requiring chemotherapy.

(2) Intervention and comparison: IPC vs. PICC.

(3) Outcomes: Complications, cost, impact on chemotherapy, and assessment of quality of life (QOF).

(4) Study design: RCTs.

The following articles were excluded: articles without initial data (see data extraction for details), meta-analyses, conference articles, case reports, and reviews. Different articles that focused on the same trial were included if they contained different outcomes; however, when analyzing the same outcome, only the most recent data were used.

### Data extraction

Two independent investigators extracted the following data: study characteristics (publication date, country, etc.), participant characteristics (cancer type, age, etc.), complications (thrombosis, infection, etc.), impact on chemotherapy (chemotherapy discontinuation, chemotherapy delay, etc.), cost (total cost, cost per catheter day, etc.), and QOL assessments (global health status, functional scales, etc.). Any disagreements were resolved through re-evaluation.

### Outcome assessments

The QOL of patients was assessed at post-implantation, mid-treatment, and end-treatment stages using the EORTC QLQ-C30 and EQ-5D scales 13,14. Complications were analyzed based on either the number of patients or catheter days. Thrombosis and infection were further categorized and analyzed according to their specific types. Thrombosis, as assessed in our study, included both deep vein thrombosis and catheter-related thrombosis. These conditions are critical complications for catheterized patients as they may lead to catheter occlusion, necessitate catheter removal, and increase the risk of further thromboembolic events. Infections, primarily CRIs, were evaluated in terms of both localized (e.g., pocket infections or exit-site infections) and systemic infections. Localized infections involve bacterial colonization around the catheter insertion site, which can lead to pain and localized inflammation, while systemic infections can progress to bacteremia or sepsis.

### Quality assessment for included studies

The quality of the RCTs was assessed using the Jadad scale, which allocates up to 7 points based on randomization, allocation concealment, double blinding, and handling of withdrawals and dropouts, with scores of 4 or higher indicating high quality 15. The quality of the results was assessed using the GRADE approach 16.

### Statistical analysis

Data pooling was analyzed using RevMan 5.3 and STATA 12.0. Continuous variables were analyzed using mean difference (MD), survival outcomes with hazard ratios (HR), and dichotomous variables with pooled risk ratios (RR). All effect sizes were presented with 95% confidence intervals (CI). Heterogeneity was assessed using the *I*² statistic and χ² test. Significant heterogeneity (*I*² > 50% or P < 0.1) warranted the use of a random-effects model; otherwise, a fixed-effects model was applied. Funnel plots were used to assess publication bias. Statistical significance was indicated by p < 0.05. (PROSPERO ID: CRD42024583534).

## Results

### Search results

**Figure 1:**
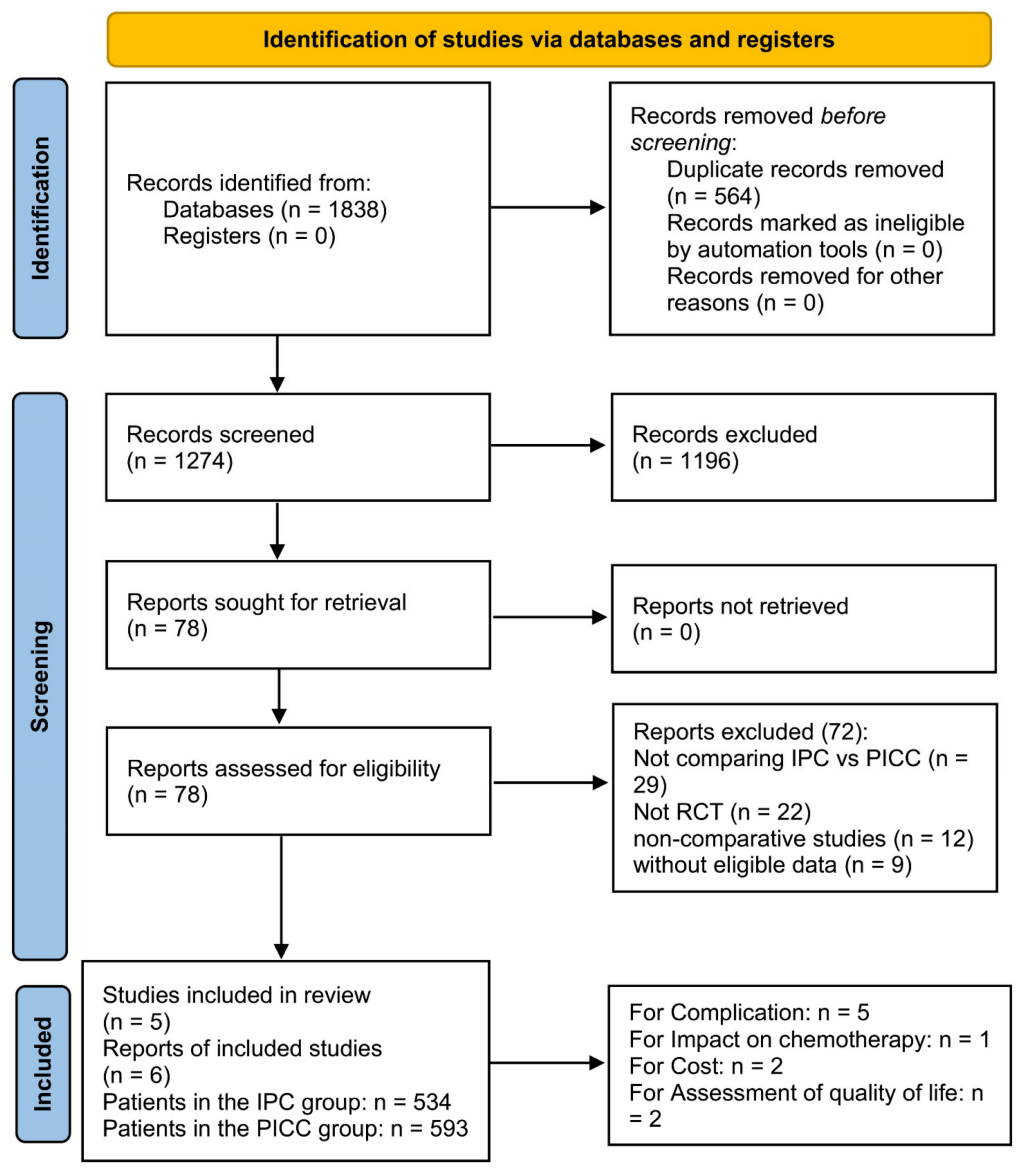
Flow chart.

Initially, 1274 studies were identified, and six papers based on five RCTs (IPC group: 534 patients; PICC group: 593 patients) were included in the analysis (**Figure 1**) 17-22. **Table 1** presents the baseline characteristics. Three studies were conducted in Europe, one in Canada, and one in Australia. All studies were rated as high quality based on the Jadad scale and Cochrane Risk Assessment (**Table S2**, **Figure S1**). However, all outcomes were rated as medium to high quality according to the GRADE system (**Table S3**).

### Complications assessment according to patients

**Figure 2:**
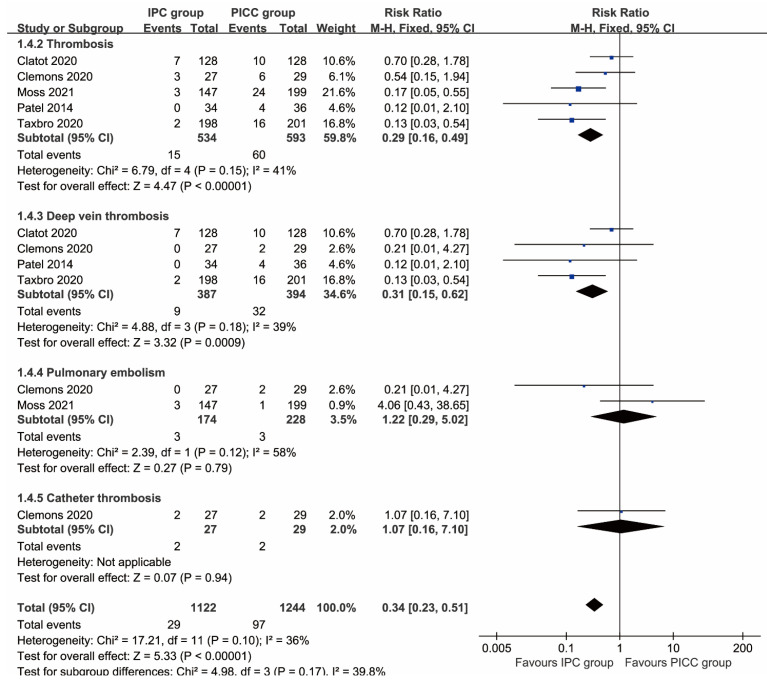
Forest plots of thrombosis (total thrombosis, deep vein thrombosis, pulmonary embolism, and catheter thrombosis) according to patients.

**Figure 3:**
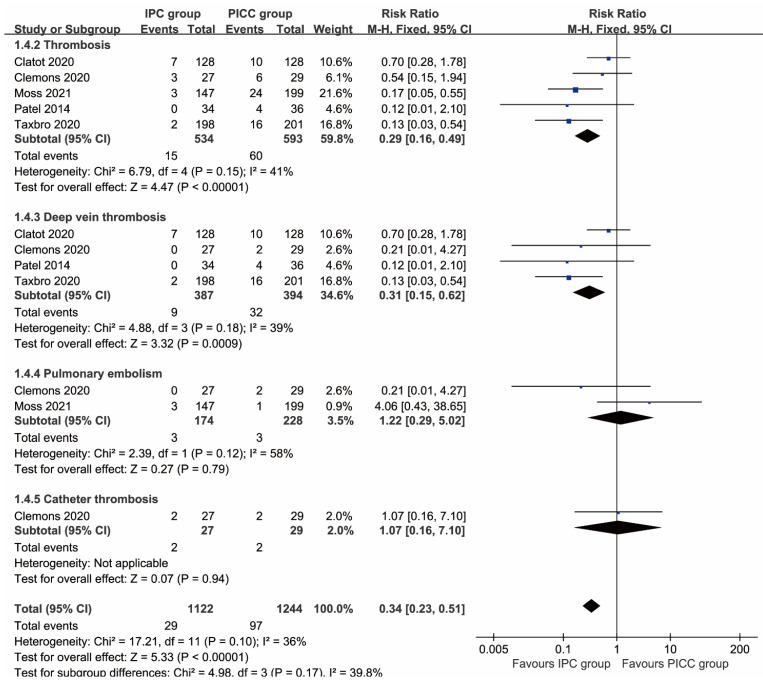
Forest plots of infection (total infection, sepsis, and pocket infection/exit-site infection without septicaemia) according to patients.

More total complications (RR: 0.51 [0.26, 0.99], P=0.03), including thrombosis (RR: 0.29 [0.16, 0.49], P<0.00001), deep vein thrombosis (RR: 0.31 [0.15, 0.62], P=0.0009), implantation failure (RR: 0.35 [0.15, 0.81], P=0.01), unplanned catheter removal (RR: 0.57 [0.41, 0.78], P=0.0005), and local reactions (RR: 0.14 [0.03, 0.62], P=0.009) were observed in the PICC group. In contrast, more pocket infections/exit-site infections without septicemia (RR: 2.34 [1.17, 4.70], P=0.02) and pain (RR: 4.06 [1.39, 11.87], P=0.01) were found in the IPC group. The rates of pulmonary embolism, catheter thrombosis, infection, sepsis, mechanical complications, catheter disruption, spontaneous catheter migration, partial withdrawal, catheter occlusion, bleeding, and edema were similar between the two groups (**Table 2**, **Figures 2, 3**). Complication-free survival was also better in the IPC group (HR: 0.37 [0.25, 0.55], P<0.00001) (**Figure S2**).

### Complications assessment according to catheter days

More total complications (RR: 0.39 [0.32, 0.48], P<0.00001), including thrombosis (RR: 0.15 [0.04, 0.54], P<0.00001), deep vein thrombosis (RR: 0.19 [0.03, 1.25], P=0.004), implantation failure (RR: 0.18 [0.08, 0.40], P<0.0001), unplanned catheter removal (RR: 0.25 [0.17, 0.37], P<0.00001), edema (RR: 0.27 [0.08, 0.96], P=0.04), and local reactions (RR: 0.09 [0.02, 0.41], P=0.002) were observed in the PICC group. The rates of pulmonary embolism, infection, sepsis, pocket infection/exit-site infection without septicemia, mechanical complications, spontaneous catheter migration, partial withdrawal, catheter occlusion, bleeding, and pain were similar between the two groups (**Table 3**).

### The impact on chemotherapy

**Figure 4:**
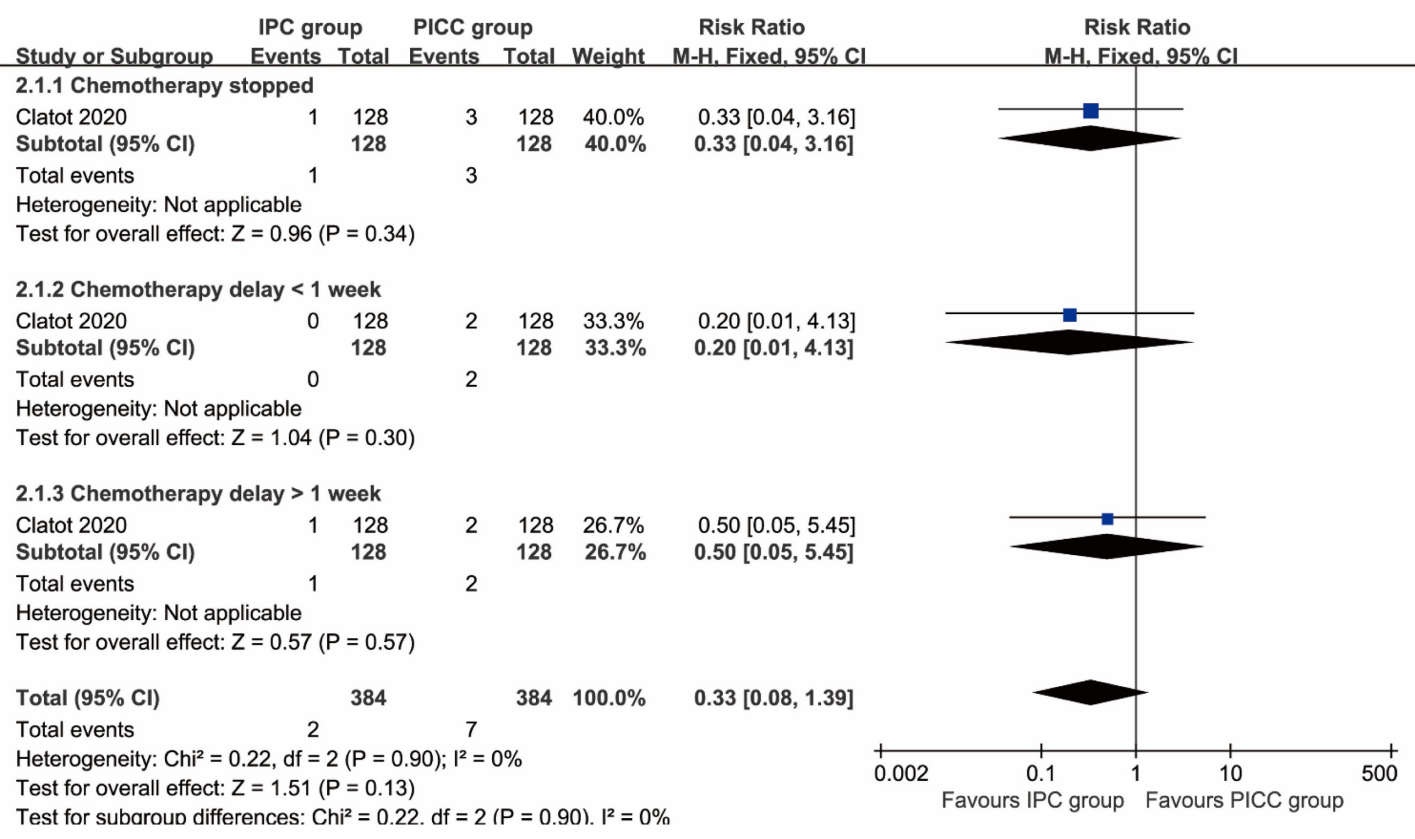
Forest plots of the impact on chemotherapy associated with IPC versus PICC.

Chemotherapy stopped (RR: 0.33 [0.04, 3.16], P=0.34), chemotherapy delay of less than 1 week (RR: 0.20 [0.01, 4.13], P=0.30), and chemotherapy delay of more than 1 week (RR: 0.50 [0.05, 5.45], P=0.57) all tended to favor the IPC group, although these differences were not statistically significant (**Figure 4**).

### Cost

The total cost (MD: 1665.00 [1595.58, 1734.42] dollars, P<0.00001) was higher in the IPC group, while the cost per catheter-day (MD: 2.82 [-14.27, 19.91] dollars, P=0.75) was similar between the two groups (**Figure S3**).

### Assessment of quality of life

QOL was assessed using the EORTC QLQ-C30. The global health status was similar between the two groups after implantation. During the post-implantation phase, social functioning (MD: -6.20 [-10.19, -2.21], P=0.002) was better in the PICC group. During mid-treatment, emotional functioning (MD: 6.70 [1.43, 11.97], P=0.01) and dyspnoea (MD: 5.70 [0.11, 11.29], P=0.05) were better in the IPC group. During end-treatment, role functioning (MD: -9.30 [-16.14, -2.46], P=0.008) and diarrhoea (MD: -5.80 [-11.41, -0.19], P=0.04) were better in the PICC group (**Table 4**).

Changes in QOL were assessed using the EORTC QLQ-C30 and EQ-5D. After implantation, the IPC group showed better improvements in global health status, physical functioning, role functioning, social functioning, fatigue, nausea and vomiting, appetite loss, constipation, financial difficulties, and health state scores. Conversely, the PICC group demonstrated better improvements in emotional functioning, cognitive functioning, pain, dyspnoea, insomnia, diarrhoea, and index value (**Table S4**).

### Sensitivity analysis

Sensitivity analyses for total complications, thrombosis, and sepsis were performed, demonstrating that excluding any individual study did not affect the reliability of the results (**Figure S4**).

### Publication bias

The analysis of complications according to catheter days, thrombosis, infection, and EORTC QLQ-C30 during the post-implantation phase revealed no evidence of publication bias (**Figure S5**).

## Discussion

The choice between IPC and PICC for chemotherapy in cancer patients remains a significant clinical debate. This controversy stems from the varying risks of complications, patient comfort, and the overall impact on treatment efficacy and quality of life. Given the high stakes of chemotherapy management, understanding which catheterization method offers superior outcomes is essential. Unlike prior meta-analyses that included retrospective studies, our study synthesizes data exclusively from RCTs, ensuring the highest evidence level and minimizing bias. This methodological strength significantly enhances the reliability of our findings and provides a more definitive comparison of IPC and PICC. Notably, our results indicate that IPC may be a safer and more comfortable option, with fewer complications and a potentially more favorable impact on chemotherapy administration, albeit at a higher overall cost.

Our analysis revealed significant differences in the complication profiles of IPC and PICC, favoring the use of IPC in several key aspects. Specifically, the PICC group exhibited a higher incidence of total complications, including thrombosis, deep vein thrombosis, implantation failure, unplanned catheter removal, and local reactions. These findings align with previous studies that have highlighted the increased risk of venous thromboembolism (VTE) associated with PICC, particularly in patients undergoing chemotherapy 23,24. The higher thrombosis rates in PICC can be attributed to the smaller lumen size and longer catheter length, which contribute to stasis and endothelial injury-key factors in thrombogenesis 25. Moreover, the ease of PICC placement, often done without fluoroscopic guidance, may result in suboptimal positioning, further increasing the risk of complications 26. In contrast, IPCs, being surgically implanted under the skin with direct access to central veins, are associated with lower thrombosis rates and fewer CRIs, as they are accessed less frequently and have a reduced risk of bacterial colonization 27,28. However, the IPC group was not without its complications, showing a higher incidence of pocket infections/exit-site infections and pain. These localized infections, while serious, are typically less life-threatening than the systemic complications more common with PICC 29.

The impact of catheter choice on chemotherapy delivery is critical, as interruptions in treatment can directly affect patient outcomes. Our study found that chemotherapy discontinuation, delays of less than one week, and delays of more than one week all tended to favor the IPC group, though these differences were not statistically significant. This trend suggests that IPC may provide a more reliable venous access route, reducing the likelihood of catheter-related issues that could disrupt chemotherapy 30. Previous research has shown that IPC, due to their stability and lower maintenance requirements, are less likely to cause treatment interruptions compared to PICC 31. This reliability is particularly important for patients receiving regimens that are highly thrombogenic or for those with a history of venous access difficulties 32. Although the differences in our analysis did not reach statistical significance, the consistent trend toward better outcomes with IPC underscores its potential advantage in maintaining uninterrupted chemotherapy, which is crucial for achieving optimal therapeutic results.

QOL is a crucial consideration when evaluating the effectiveness of venous access devices. Our study assessed QOL using the EORTC QLQ-C30 and EQ-5D scales at various stages of treatment and found that global health status was similar between the IPC and PICC groups during post-implantation, mid-treatment, and end-treatment periods. However, the decline in global health status post-implantation was smaller in the IPC group compared to the PICC group, suggesting that IPC may be less disruptive to patients' overall well-being 33. This finding aligns with previous studies indicating that IPCs, being fully implanted and less intrusive, contribute to higher patient satisfaction and better adherence to treatment 34. In contrast, PICC, which are visible and require regular maintenance, may lead to increased anxiety and discomfort, negatively affecting the patient's QOL 35. Additionally, our analysis of specific QOL domains revealed that emotional functioning and dyspnea were better in the IPC group during mid-treatment, further supporting the notion that IPC may offer a more favorable experience for patients undergoing prolonged chemotherapy.

While our study provides important insights, it is not without limitations. First, the inclusion of only a limited number of RCTs may restrict the generalizability of our findings. Despite rigorous selection criteria, the small sample size and heterogeneity of the included studies may have introduced bias. Second, the quality of evidence for some outcomes was rated as medium by the GRADE system, which may affect the robustness of our conclusions. Third, cost analyses were limited by the lack of standardized reporting across studies, making it difficult to draw definitive conclusions about the economic impact of IPC versus PICC. Fourth, the sample size across the included studies varied, which may impact the statistical power and generalizability of the results. Finally, geographical differences and variations in clinical practice may limit the applicability of our conclusions to all patient populations. Differences in catheter management protocols, healthcare infrastructure, and patient demographics across regions could influence complication rates and patient experiences, thus affecting the broader generalizability of IPC and PICC comparisons. Future studies with larger, more diverse populations could provide more robust evidence and help validate our findings in varied clinical contexts.

## Conclusions

IPC may be a safer and more comfortable option for cancer patients requiring chemotherapy compared to PICC. IPC is associated with fewer complications and may provide a more reliable venous access route, thereby reducing the risk of chemotherapy interruptions. While both devices had a similar impact on overall quality of life, IPC appeared to be less disruptive to patients' well-being. However, these findings should be interpreted with caution due to the study's limitations, and further high-quality research is needed to confirm these results.

## Supplementary Material

Supplementary figures and tables.

## Figures and Tables

**Table 1:** Baseline characteristics of the included studies.

Study	ID	Country	Period (year)	Groups	Number of patients	Sex (M/F)	Age (Mean, year)	Cancer type	Quality
Clatot 2020 17	NCT02095743	France	2014.02-2018.05	IPC	128	0/128	56	Breast cancer	6
				PICC	128	0/128	57.5		
Clemons 2020 18	NCT02632435	Canada	2016.03-2018.03	IPC	27	0/27	54	Breast cancer	8
				PICC	29	0/29	52		
Moss 2021 19	ISRCTN44504648	UK	2013.11-2018.02-	IPC	147	66/81	61	Haematological malignancy and Solid tumour	6
				PICC	199	92/107	61		
Patel 2014 20	-	Australia	2004.12-2010.01	IPC	34	19/15	60	Haematological malignancy and Solid tumour	4
				PICC	36	17/19	59		
Taxbro 2020 21, Taxbro 2020 22	NCT01971021	Sweden	2013.03-2017.02	IPC	198	83/115	65	Haematological malignancy and Solid tumour	5
				PICC	201	91/110	66		

**Abbreviations:** ID: Identity document; IPC: Implantable Port Catheter; M/F: Male/Female; PICC: Peripherally Inserted Central Catheter.

**Table 2:** Complications assessment according to patients.

Complications	Studies involved	IPC			PICC		Risk ratio [95% CI]	P-value
		Event/total	%		Event/total	%		
Total	5	175/534	32.77%		269/593	45.36%	0.79 [0.63, 0.98]	0.03
Thrombosis	5	15/534	2.81%		60/593	10.12%	0.29 [0.16, 0.49]	< 0.00001
Deep vein thrombosis	4	9/387	2.33%		32/394	8.12%	0.31 [0.15, 0.62]	0.0009
Pulmonary embolism	2	3/174	1.72%		3/228	1.32%	1.22 [0.29, 5.02]	0.79
Catheter thrombosis	1	2/27	7.41%		2/29	6.90%	1.07 [0.16, 7.10]	0.94
Infection	3	27/309	8.74%		21/363	5.79%	1.63 [0.96, 2.78]	0.07
Sepsis	4	13/500	2.60%		12/557	2.15%	1.31 [0.62, 2.77]	0.48
Pocket infection/exit-site infection without septicaemia	4	24/500	4.80%		11/557	1.97%	2.34 [1.17, 4.70]	0.02
Mecanical complication	2	12/326	3.68%		9/329	2.74%	2.12 [0.15, 29.22]	0.58
Implantation failure	3	7/473	1.48%		25/528	4.73%	0.35 [0.15, 0.81]	0.01
Catheter disruption	1	1/34	2.94%		2/36	5.56%	0.53 [0.05, 5.57]	0.6
Spontaneous catheter migration	2	0/162	0.00%		4/164	2.44%	0.21 [0.02, 1.73]	0.15
Unplanned catheter removal	3	42/302	13.91%		95/356	26.69%	0.57 [0.41, 0.78]	0.0005
Partial withdrawal	1	0/128	0.00%		1/128	0.78%	0.33 [0.01, 8.11]	0.5
Catheter occlusion	3	3/360	0.83%		19/365	5.21%	0.31 [0.02, 3.92]	0.36
Bleeding	1	0/128	0.00%		1/128	0.78%	0.33 [0.01, 8.11]	0.5
Oedema	2	3/326	0.92%		7/329	2.13%	0.47 [0.13, 1.67]	0.24
Local reaction	1	2/128	1.56%		14/128	10.94%	0.14 [0.03, 0.62]	0.009
Pain	2	16/162	9.88%		4/164	2.44%	4.06 [1.39, 11.87]	0.01

**Abbreviations:** CI: Confidence Interval; IPC: Implantable Port Catheter; PICC: Peripherally Inserted Central Catheter.

**Table 3:** Complications assessment according to catheter days.

Complications	Studies involved	IPC			PICC		Risk ratio [95% CI]	P-value
		Event/total	%		Event/total	%		
Total	3	148/73127	0.202%		233/43553	0.535%	0.39 [0.32, 0.48]	< 0.00001
Thrombosis	3	12/73127	0.016%		50/43553	0.115%	0.15 [0.04, 0.54]	< 0.00001
Deep vein thrombosis	2	9/61227	0.015%		26/36068	0.072%	0.19 [0.03, 1.25]	0.004
Pulmonary embolism	1	3/11900	0.025%		1/7485	0.013%	1.89 [0.20, 18.14]	0.58
Infection	2	27/29436	0.092%		20/18877	0.106%	0.85 [0.48, 1.52]	0.59
Sepsis	3	12/73127	0.016%		10/43553	0.023%	0.74 [0.33, 1.69]	0.48
Pocket infection/exit-site infection without septicaemia	3	19/73127	0.026%		10/43553	0.023%	1.10 [0.51, 2.34]	0.81
Mecanical complication	2	12/61227	0.020%		9/36068	0.025%	1.30 [0.08, 21.34]	0.86
Implantation failure	3	7/73127	0.010%		25/43553	0.057%	0.18 [0.08, 0.40]	< 0.0001
Spontaneous catheter migration	1	0/17536	0.000%		2/11392	0.018%	0.13 [0.01, 2.71]	0.19
Unplanned catheter removal	2	37/29436	0.126%		92/18877	0.487%	0.25 [0.17, 0.37]	< 0.00001
Partial withdrawal	1	0/17536	0.000%		1/11392	0.009%	0.22 [0.01, 5.32]	0.35
Catheter occlusion	1	0/17536	0.000%		1/11392	0.009%	0.22 [0.01, 5.32]	0.35
Bleeding	1	0/17536	0.000%		1/11392	0.009%	0.22 [0.01, 5.32]	0.35
Oedema	2	3/61227	0.005%		7/36068	0.019%	0.27 [0.08, 0.96]	0.04
Local reaction	1	2/17536	0.011%		14/11392	0.123%	0.09 [0.02, 0.41]	0.002
Pain	1	12/17536	0.068%		3/11392	0.026%	2.60 [0.73, 9.21]	0.14

**Abbreviations:** CI: Confidence Interval; IPC: Implantable Port Catheter; PICC: Peripherally Inserted Central Catheter.

**Table 4:** EORTC QLQ-C30 assessment between IPC and PICC during post implantation, mid treatment, and end treatment.

EORTC QLQ-C30	Post implantation			Mid treatment			End treatment	
	MD [95% CI]	P-value		MD [95% CI]	P-value		MD [95% CI]	P-value
Global health status	0.35 [-2.81, 3.52]	0.83		4.80 [-0.04, 9.64]	0.05		-3.40 [-8.35, 1.55]	0.18
Functional scales								
Physical functioning	-2.20 [-4.95, 0.55]	0.12		-0.50 [-3.87, 2.87]	0.77		-1.30 [-5.96, 3.36]	0.58
Role functioning	-2.00 [-6.27, 2.27]	0.36		-0.90 [-6.92, 5.12]	0.77		-9.30 [-16.14, -2.46]	0.008
Emotional functioning	0.50 [-4.87, 5.87]	0.86		6.70 [1.43, 11.97]	0.01		-3.20 [-8.81, 2.41]	0.26
Cognitive functioning	0.30 [-3.12, 3.72]	0.86		0.10 [-4.79, 4.99]	0.97		-5.80 [-11.00, -0.60]	0.03
Social functioning	-6.20 [-10.19, -2.21]	0.002		0.30 [-4.79, 5.39]	0.91		-4.60 [-10.80, 1.60]	0.15
Symptoms scales								
Fatigue	4.80 [-0.09, 9.69]	0.05		-1.70 [-7.47, 4.07]	0.56		3.80 [-2.87, 10.47]	0.26
Nausea and vomiting	-0.20 [-2.40, 2.00]	0.86		-2.80 [-8.78, 3.18]	0.36		-1.70 [-5.20, 1.80]	0.34
Pain	2.00 [-2.91, 6.91]	0.42		-1.70 [-6.02, 2.62]	0.44		1.10 [-4.83, 7.03]	0.72
Dyspnoea	-3.80 [-8.06, 0.46]	0.08		5.70 [0.11, 11.29]	0.05		6.30 [-0.61, 13.21]	0.07
Insomnia	1.30 [-6.04, 8.64]	0.73		3.00 [-4.34, 10.34]	0.42		-4.30 [-12.26, 3.66]	0.29
Appetite loss	0.40 [-4.92, 5.72]	0.88		0.80 [-6.82, 8.42]	0.84		-5.30 [-12.13, 1.53]	0.13
Constipation	-4.10 [-8.76, 0.56]	0.08		-4.00 [-10.18, 2.18]	0.2		-3.20 [-10.11, 3.71]	0.36
Diarrhoea	0.60 [-2.62, 3.82]	0.72		1.20 [-4.78, 7.18]	0.69		-5.80 [-11.41, -0.19]	0.04
Financial difficulties	1.10 [-3.86, 6.06]	0.66		-3.80 [-9.76, 2.16]	0.21		0.80 [-5.62, 7.22]	0.81

**Abbreviations:** CI: Confidence Interval; EORTC QLQ-C30: European Organisation for Research and Treatment of Cancer Quality of Life Questionnaire Core 30; IPC: Implantable Port Catheter; MD: Mean Difference; PICC: Peripherally Inserted Central Catheter.

## Abbreviations

CI: Confidence Interval
CRIs: Catheter-Related Infections
EORTC QLQ-C30: European Organisation for Research and Treatment of Cancer Quality of Life Questionnaire Core 30
EQ-5D: EuroQol 5-Dimension Scale
GRADE: Grading of Recommendations Assessment, Development and Evaluation
HR: Hazard ratios
ID: Identity document
IPC: Implantable Port Catheter
MD: Mean Difference
M/F: Male/Female
NOS: Newcastle-Ottawa Scale
PICC: Peripherally Inserted Central Catheter
PRISMA: Preferred Reporting Items for Systematic Reviews and Meta-Analyses
QOL: Quality of Life
RCT: Randomized Controlled Trial
RR: Risk Ratio
VTE: Venous Thromboembolism
