# Supplementary material for: Implantable Port Catheters versus Peripherally Inserted Central Catheters for Cancer Patients Requiring Chemotherapy: An RCT-Based Meta-Analysis
Source: J Cancer. 2025 Jan 6;16(4):1127–36. doi: 10.7150/jca.103631 (PMC11786043; doi:10.7150/jca.103631)
Supplement: Supplementary file 1 — Supplementary figures and tables. [file jcav16p1127s1.pdf]

Supplementary Data

Figure S1 Cochrane Risk Assessment.

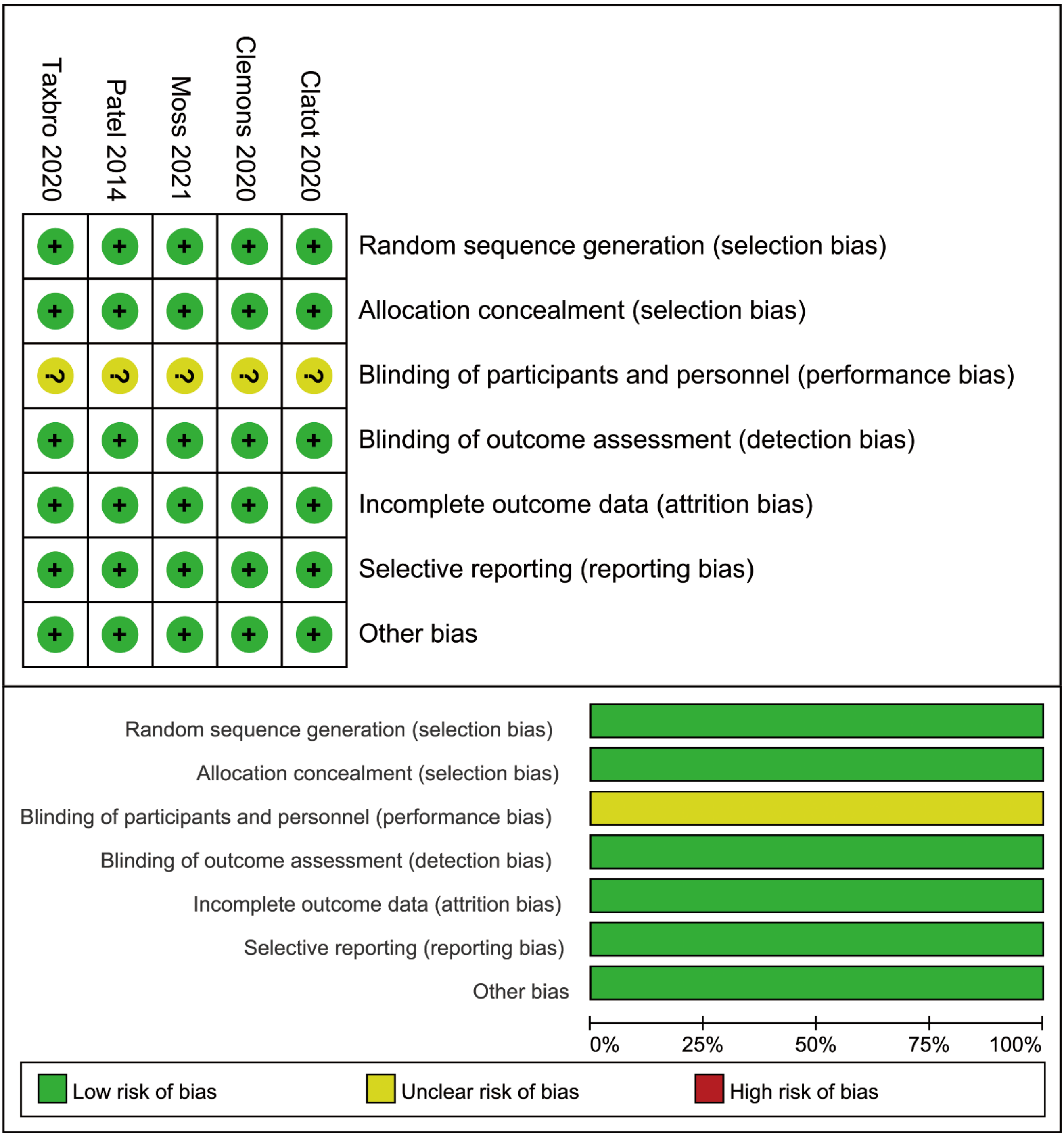

Figure S2 Forest plots of complication-free survival associated with IPC versus PICC.

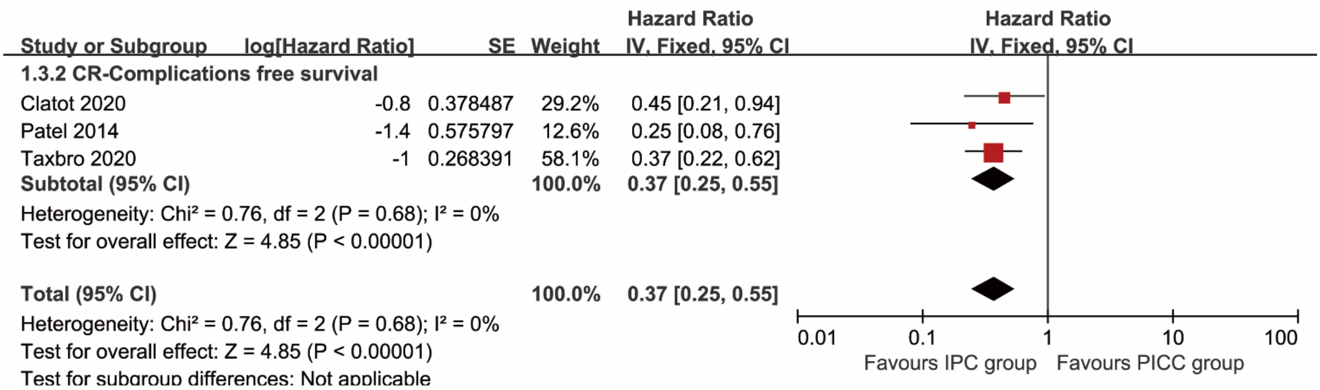

Figure S3 Forest plots of cost associated with IPC versus PICC.

Study or Subgroup	IPC group Mean	IPC group SD	IPC group Total	PICC group Mean	PICC group SD	PICC group Total	Weight	Mean Difference IV, Random, 95% CI
**5.3.1 Total cost**								
Moss 2021	2,706	412	147	1,041	141	199	26.0%	1665.00 [1595.58, 1734.42]
**Subtotal (95% CI)**			**147**			**199**	**26.0%**	**1665.00 [1595.58, 1734.42]**
Heterogeneity: Not applicable								
Test for overall effect: Z = 47.01 (P < 0.00001)								
**5.3.2 Cost per catheter-day**								
Moss 2021	37.5	9.5	147	43.4	11	199	37.0%	-5.90 [-8.07, -3.73]
Patel 2014	23.63	5.5	34	12.09	4	36	37.0%	11.54 [9.28, 13.80]
**Subtotal (95% CI)**			**181**			**235**	**74.0%**	**2.82 [-14.27, 19.91]**
Heterogeneity: Tau² = 150.80; Chi² = 118.99, df = 1 (P < 0.00001); I² = 99%								
Test for overall effect: Z = 0.32 (P = 0.75)								
**Total (95% CI)**			**328**			**434**	**100.0%**	**434.93 [370.06, 499.80]**
Heterogeneity: Tau² = 2959.33; Chi² = 2321.01, df = 2 (P < 0.00001); I² = 100%								
Test for overall effect: Z = 13.14 (P < 0.00001)								
Test for subgroup differences: Chi² = 2076.30, df = 1 (P < 0.00001), I² = 100.0%								

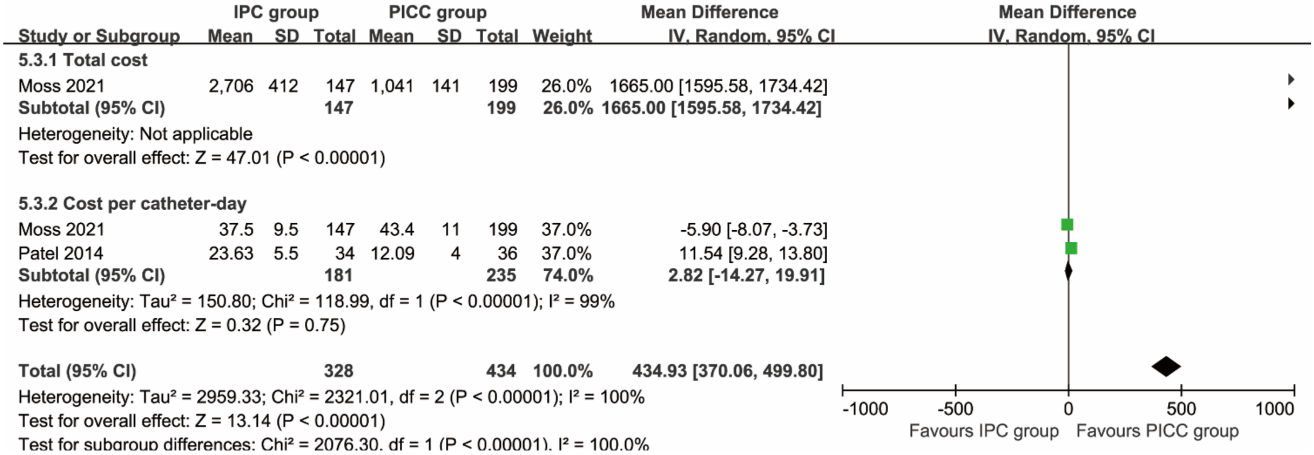

Figure S4 Sensitivity analysis of total complications (A), thrombosis (B), and sepsis (C).

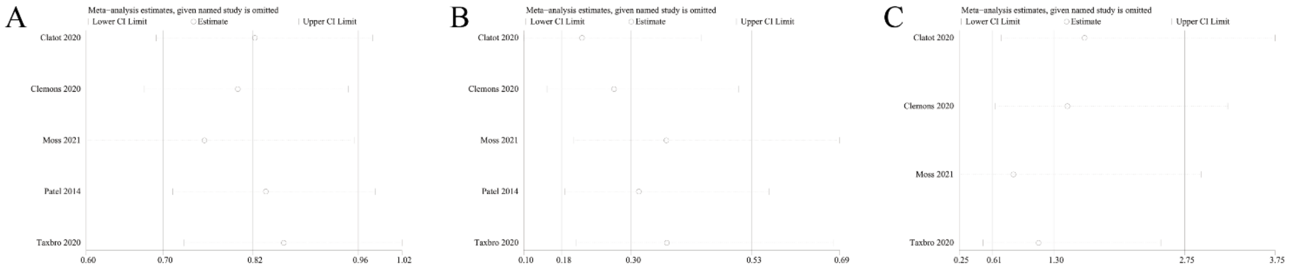

A

Study omitted	Estimate	[95% Conf.	Interval]
Clatot 2020	.82284784	.69140238	.97928298
Clemons 2020	.80005646	.67583042	.94711673
Moss 2021	.75585824	.5983212	.95487463
Patel 2014	.83750242	.71373808	.98272794
Taxbro 2020	.86130607	.72838271	1.0184867
Combined	.82029023	.70062378	.9603957

B

Study omitted	Estimate	[95% Conf.	Interval]
Clatot 2020	.21290515	.10424736	.43481773
Clemons 2020	.27287504	.14752923	.5047189
Moss 2021	.3702268	.19761029	.69362724
Patel 2014	.31902158	.18117964	.56173402
Taxbro 2020	.37128824	.20230792	.68141156
Combined	.3045854	.17505086	.5299732

C

Study omitted	Estimate	[95% Conf.	Interval]
Clatot 2020	1.6369864	.71409667	3.7526073
Clemons 2020	1.4467592	.6483348	3.2284434
Moss 2021	.84941459	.2461336	2.9313557
Taxbro 2020	1.1277236	.51174045	2.4851668
Combined	1.2981204	.61260877	2.7507224

Table S1 Search strategy.

**PubMed**<br>The database was searched on August 1, 2024, n=73.<br>Search Strategy:<br>#1 Search: PICC[Title/Abstract] OR Peripherally inserted central venous catheter[Title/Abstract] OR Peripherally inserted central catheter[Title/Abstract] OR Percutaneous indwelling central catheter[Title/Abstract] OR Central venous catheter[Title/Abstract] Sort by: Most Recent n = 11753<br>#2 Search: IPC[Title/Abstract] OR Implantable port catheter[Title/Abstract] OR Implantable access port[Title/Abstract] OR Central venous port access system[Title/Abstract] OR Vascular access device[Title/Abstract] OR TIVAD[Title/Abstract] Sort by: Most Recent n = 6588<br>#1 and #2 n = 73
**Web of Science**<br>The database was searched on August 1, 2024, n=960.<br>Search Strategy:<br>(PICC(Abstract) OR Peripherally inserted central venous catheter(Abstract) OR Peripherally inserted central catheter(Abstract) OR Percutaneous indwelling central catheter(Abstract) OR Central venous catheter(Abstract)) AND (IPC(Abstract) OR Implantable port catheter(Abstract) OR Implantable access port(Abstract) OR Central venous port access system(Abstract) OR Vascular access device(Abstract) OR TIVAD(Abstract))
**EMBASE**<br>The database was searched on August 1, 2024, n=153.<br>Search Strategy:<br>(PICC:ti,ab,kw OR Peripherally inserted central venous catheter:ti,ab,kw OR Peripherally inserted central catheter:ti,ab,kw OR Percutaneous indwelling central catheter:ti,ab,kw OR Central venous catheter:ti,ab,kw) AND (IPC:ti,ab,kw OR Implantable port catheter:ti,ab,kw OR Implantable access port:ti,ab,kw OR Central venous port access system:ti,ab,kw OR Vascular access device:ti,ab,kw OR TIVAD:ti,ab,kw)
**Cochrane Library**<br>The database was searched on August 1, 2024, n=26.<br>Search Strategy:<br>(PICC OR Peripherally inserted central venous catheter OR Peripherally inserted central catheter OR Percutaneous indwelling central catheter OR Central venous catheter) in Title Abstract Keyword AND (IPC OR Implantable port catheter OR Implantable access port OR Central venous port access system OR Vascular access device OR TIVAD) in Title Abstract Keyword
**ScienceDirect**<br>The database was searched on August 1, 2024, n=397.<br>Search Strategy:<br>Title, abstract, keywords: ((“PICC” OR “Peripherally inserted central venous catheter” OR “Peripherally inserted central catheter” OR “Percutaneous indwelling central catheter” OR “Central venous catheter”) AND (“IPC” OR “Implantable port catheter” OR “Implantable access port” OR “Central venous port access system” OR “Vascular access device” OR “TIVAD”)
**Scopus**<br>The database was searched on August 1, 2024, n=131.

Search Strategy:

(TITLE-ABS-KEY (PICC OR Peripherally inserted central venous catheter OR Peripherally inserted central catheter OR Percutaneous indwelling central catheter OR Central venous catheter) AND TITLE-ABS-KEY (IPC OR Implantable port catheter OR Implantable access port OR Central venous port access system OR Vascular access device OR TIVAD)

Note: The combined text and medical subject heading (MeSH) terms used were: "Peripherally inserted central venous catheter" and "Implantable port catheter".

Table S2 Quality assessments of randomized clinical trials according to Jadad scale.

Study	Randomization	Concealment of allocation	Double blinding	Withdrawals and dropouts	Quality (score)
Clatot 2020 [17]	**	**	*	*	6
Clemons 2020 [18]	**	*	*	*	5
Moss 2021 [19]	**	**	*	*	6
Patel 2014 [20]	**	*	*	*	4
Taxbro 2020 [21], Taxbro 2020 [22]	**	*	*	*	5

Table S3 GRADE quality assessment by therapeutic strategy and study design for the outcomes.

Primary outcomes	No. of Studies	No. of Participants		Differences (95%CI) [a]	Quality Assessment					Quality
		IPC	PICC		Risk of Bias[b]	Inconsistency	Indirectness	Imprecision	Publication Bias[c]	
**Complication-free survival**	3	360	365	0.37 [0.25, 0.55]	Low	No inconsistency	No indirectness	No imprecision	Unlikely	High
**Complications assessed according to patients**										
Total	5	175/534	269/593	0.79 [0.63, 0.98]	Low	Serious (-1)	No indirectness	No imprecision	Unlikely	Medium
Thrombosis	5	15/534	60/593	0.29 [0.16, 0.49]	Low	No inconsistency	No indirectness	No imprecision	Unlikely	High
Deep vein thrombosis	4	9/387	32/394	0.31 [0.15, 0.62]	Low	No inconsistency	No indirectness	No imprecision	Unlikely	High
Pulmonary embolism	2	3/174	3/228	1.22 [0.29, 5.02]	Low	No inconsistency	No indirectness	No imprecision	Unlikely	High
Catheter thrombosis	1	2/27	2/29	1.07 [0.16, 7.10]	Low	No inconsistency	No indirectness	No imprecision	Unlikely	High
Infection	3	27/309	21/363	1.63 [0.96, 2.78]	Low	No inconsistency	No indirectness	No imprecision	Unlikely	High
Sepsis	4	13/500	12/557	1.31 [0.62, 2.77]	Low	No inconsistency	No indirectness	No imprecision	Unlikely	High
Pocket infection/exit-site infection without septicaemia	4	24/500	11/557	2.34 [1.17, 4.70]	Low	No inconsistency	No indirectness	No imprecision	Unlikely	High
Mecanical complication	2	12/326	9/329	2.12 [0.15, 29.22]	Low	Serious (-1)	No indirectness	No imprecision	Unlikely	Medium
Implantation failure	3	7/473	25/528	0.35 [0.15, 0.81]	Low	No inconsistency	No indirectness	No imprecision	Unlikely	High
Catheter disruption	1	1/34	2/36	0.53 [0.05, 5.57]	Low	No inconsistency	No indirectness	No imprecision	Unlikely	High
Spontaneous catheter migration	2	0/162	4/164	0.21 [0.02, 1.73]	Low	No inconsistency	No indirectness	No imprecision	Unlikely	High
Unplanned catheter Removal	3	42/302	95/356	0.57 [0.41, 0.78]	Low	No inconsistency	No indirectness	No imprecision	Unlikely	High
Partial withdrawal	1	0/128	1/128	0.33 [0.01, 8.11]	Low	No inconsistency	No indirectness	No imprecision	Unlikely	High
Catheter occlusion	3	3/360	19/365	0.31 [0.02, 3.92]	Low	Serious (-1)	No indirectness	No imprecision	Unlikely	Medium
Bleeding	1	0/128	1/128	0.33 [0.01, 8.11]	Low	No inconsistency	No indirectness	No imprecision	Unlikely	High
Oedema	2	3/326	7/329	0.47 [0.13, 1.67]	Low	No inconsistency	No indirectness	No imprecision	Unlikely	High
Local reaction	1	2/128	14/128	0.14 [0.03, 0.62]	Low	No inconsistency	No indirectness	No imprecision	Unlikely	High
Pain	2	16/162	4/164	4.06 [1.39, 11.87]	Low	No inconsistency	No indirectness	No imprecision	Unlikely	High
**Complications assessed according to catheter days**										
Total	3	148/73127	233/43553	0.39 [0.32, 0.48]	Low	No inconsistency	No indirectness	No imprecision	Unlikely	High
Thrombosis	3	12/73127	50/43553	0.15 [0.04, 0.54]	Low	Serious (-1)	No indirectness	No imprecision	Unlikely	Medium
Deep vein thrombosis	2	9/61227	26/36068	0.19 [0.03, 1.25]	Low	Serious (-1)	No indirectness	No imprecision	Unlikely	Medium
Pulmonary embolism	1	3/11900	1/7485	1.89 [0.20, 18.14]	Low	No inconsistency	No indirectness	No imprecision	Unlikely	High
Infection	2	27/29436	20/18877	0.85 [0.48, 1.52]	Low	No inconsistency	No indirectness	No imprecision	Unlikely	High
Sepsis	3	12/73127	10/43553	0.74 [0.33, 1.69]	Low	No inconsistency	No indirectness	No imprecision	Unlikely	High

Pocket infection/exit-site infection without septicaemia	3	19/73127	10/43553	1.10 [0.51, 2.34]	Low	No inconsistency	No indirectness	No imprecision	Unlikely	High
Mecanical complication	2	12/61227	9/36068	1.30 [0.08, 21.34]	Low	Serious (-1)	No indirectness	No imprecision	Unlikely	Medium
Implantation failure	3	7/73127	25/43553	0.18 [0.08, 0.40]	Low	No inconsistency	No indirectness	No imprecision	Unlikely	High
Spontaneous catheter migration	1	0/17536	2/11392	0.13 [0.01, 2.71]	Low	No inconsistency	No indirectness	No imprecision	Unlikely	High
Unplanned catheter Removal	2	37/29436	92/18877	0.25 [0.17, 0.37]	Low	No inconsistency	No indirectness	No imprecision	Unlikely	High
Partial withdrawal	1	0/17536	1/11392	0.22 [0.01, 5.32]	Low	No inconsistency	No indirectness	No imprecision	Unlikely	High
Catheter occlusion	1	0/17536	1/11392	0.22 [0.01, 5.32]	Low	No inconsistency	No indirectness	No imprecision	Unlikely	High
Bleeding	1	0/17536	1/11392	0.22 [0.01, 5.32]	Low	No inconsistency	No indirectness	No imprecision	Unlikely	High
Oedema	2	3/61227	7/36068	0.27 [0.08, 0.96]	Low	No inconsistency	No indirectness	No imprecision	Unlikely	High
Local reaction	1	2/17536	14/11392	0.09 [0.02, 0.41]	Low	No inconsistency	No indirectness	No imprecision	Unlikely	High
Pain	1	12/17536	3/11392	2.60 [0.73, 9.21]	Low	No inconsistency	No indirectness	No imprecision	Unlikely	High
**Impact on chemotherapy**										
Chemotherapy stopped	1	128	128	0.33 [0.04, 3.16]	Low	No inconsistency	No indirectness	No imprecision	Unlikely	High
Chemotherapy delay < 1 week	1	128	128	0.20 [0.01, 4.13]	Low	No inconsistency	No indirectness	No imprecision	Unlikely	High
Chemotherapy delay > 1 week	1	128	128	0.50 [0.05, 5.45]	Low	No inconsistency	No indirectness	No imprecision	Unlikely	High
**Cost**										
Total cost	1	147	199	1665.00 [1595.58, 1734.42]	Low	No inconsistency	No indirectness	No imprecision	Unlikely	High
Cost per catheter-day	2	181	235	2.82 [-14.27, 19.91]	Low	No inconsistency	No indirectness	No imprecision	Unlikely	High
**EORTC QLQ-C30**										
**Post implantation**										
Global health status	2	275	327	0.35 [-2.81, 3.52]	Low	No inconsistency	No indirectness	No imprecision	Unlikely	High
Physical functioning	1	128	128	-2.20 [-4.95, 0.55]	Low	No inconsistency	No indirectness	No imprecision	Unlikely	High
Role functioning	1	128	128	-2.00 [-6.27, 2.27]	Low	No inconsistency	No indirectness	No imprecision	Unlikely	High
Emotional functioning	1	128	128	0.50 [-4.87, 5.87]	Low	No inconsistency	No indirectness	No imprecision	Unlikely	High
Cognitive functioning	1	128	128	0.30 [-3.12, 3.72]	Low	No inconsistency	No indirectness	No imprecision	Unlikely	High
Social functioning	1	128	128	-6.20 [-10.19, -2.21]	Low	No inconsistency	No indirectness	No imprecision	Unlikely	High
Fatigue	1	128	128	4.80 [-0.09, 9.69]	Low	No inconsistency	No indirectness	No imprecision	Unlikely	High
Nausea and vomiting	1	128	128	-0.20 [-2.40, 2.00]	Low	No inconsistency	No indirectness	No imprecision	Unlikely	High
Pain	1	128	128	2.00 [-2.91, 6.91]	Low	No inconsistency	No indirectness	No imprecision	Unlikely	High
Dyspnoea	1	128	128	-3.80 [-8.06, 0.46]	Low	No inconsistency	No indirectness	No imprecision	Unlikely	High
Insomnia	1	128	128	1.30 [-6.04, 8.64]	Low	No inconsistency	No indirectness	No imprecision	Unlikely	High
Appetite loss	1	128	128	0.40 [-4.92, 5.72]	Low	No inconsistency	No indirectness	No imprecision	Unlikely	High
Constipation	1	128	128	-4.10 [-8.76, 0.56]	Low	No inconsistency	No indirectness	No imprecision	Unlikely	High

Diarrhoea	1	128	128	0.60 [-2.62, 3.82]	Low	No inconsistency	No indirectness	No imprecision	Unlikely	High
Financial difficulties	1	128	128	1.10 [-3.86, 6.06]	Low	No inconsistency	No indirectness	No imprecision	Unlikely	High
**Mid treatment**										
Global health status	1	128	128	4.80 [-0.04, 9.64]	Low	No inconsistency	No indirectness	No imprecision	Unlikely	High
Physical functioning	1	128	128	-0.50 [-3.87, 2.87]	Low	No inconsistency	No indirectness	No imprecision	Unlikely	High
Role functioning	1	128	128	-0.90 [-6.92, 5.12]	Low	No inconsistency	No indirectness	No imprecision	Unlikely	High
Emotional functioning	1	128	128	6.70 [1.43, 11.97]	Low	No inconsistency	No indirectness	No imprecision	Unlikely	High
Cognitive functioning	1	128	128	0.10 [-4.79, 4.99]	Low	No inconsistency	No indirectness	No imprecision	Unlikely	High
Social functioning	1	128	128	0.30 [-4.79, 5.39]	Low	No inconsistency	No indirectness	No imprecision	Unlikely	High
Fatigue	1	128	128	-1.70 [-7.47, 4.07]	Low	No inconsistency	No indirectness	No imprecision	Unlikely	High
Nausea and vomiting	1	128	128	-2.80 [-8.78, 3.18]	Low	No inconsistency	No indirectness	No imprecision	Unlikely	High
Pain	1	128	128	-1.70 [-6.02, 2.62]	Low	No inconsistency	No indirectness	No imprecision	Unlikely	High
Dyspnoea	1	128	128	5.70 [0.11, 11.29]	Low	No inconsistency	No indirectness	No imprecision	Unlikely	High
Insomnia	1	128	128	3.00 [-4.34, 10.34]	Low	No inconsistency	No indirectness	No imprecision	Unlikely	High
Appetite loss	1	128	128	0.80 [-6.82, 8.42]	Low	No inconsistency	No indirectness	No imprecision	Unlikely	High
Constipation	1	128	128	-4.00 [-10.18, 2.18]	Low	No inconsistency	No indirectness	No imprecision	Unlikely	High
Diarrhoea	1	128	128	1.20 [-4.78, 7.18]	Low	No inconsistency	No indirectness	No imprecision	Unlikely	High
Financial difficulties	1	128	128	-3.80 [-9.76, 2.16]	Low	No inconsistency	No indirectness	No imprecision	Unlikely	High
**End treatment**										
Global health status	1	128	128	-3.40 [-8.35, 1.55]	Low	No inconsistency	No indirectness	No imprecision	Unlikely	High
Physical functioning	1	128	128	-1.30 [-5.96, 3.36]	Low	No inconsistency	No indirectness	No imprecision	Unlikely	High
Role functioning	1	128	128	-9.30 [-16.14, -2.46]	Low	No inconsistency	No indirectness	No imprecision	Unlikely	High
Emotional functioning	1	128	128	-3.20 [-8.81, 2.41]	Low	No inconsistency	No indirectness	No imprecision	Unlikely	High
Cognitive functioning	1	128	128	-5.80 [-11.00, -0.60]	Low	No inconsistency	No indirectness	No imprecision	Unlikely	High
Social functioning	1	128	128	-4.60 [-10.80, 1.60]	Low	No inconsistency	No indirectness	No imprecision	Unlikely	High
Fatigue	1	128	128	3.80 [-2.87, 10.47]	Low	No inconsistency	No indirectness	No imprecision	Unlikely	High
Nausea and vomiting	1	128	128	-1.70 [-5.20, 1.80]	Low	No inconsistency	No indirectness	No imprecision	Unlikely	High
Pain	1	128	128	1.10 [-4.83, 7.03]	Low	No inconsistency	No indirectness	No imprecision	Unlikely	High
Dyspnoea	1	128	128	6.30 [-0.61, 13.21]	Low	No inconsistency	No indirectness	No imprecision	Unlikely	High
Insomnia	1	128	128	-4.30 [-12.26, 3.66]	Low	No inconsistency	No indirectness	No imprecision	Unlikely	High
Appetite loss	1	128	128	-5.30 [-12.13, 1.53]	Low	No inconsistency	No indirectness	No imprecision	Unlikely	High
Constipation	1	128	128	-3.20 [-10.11, 3.71]	Low	No inconsistency	No indirectness	No imprecision	Unlikely	High
Diarrhoea	1	128	128	-5.80 [-11.41, -0.19]	Low	No inconsistency	No indirectness	No imprecision	Unlikely	High
Financial difficulties	1	128	128	0.80 [-5.62, 7.22]	Low	No inconsistency	No indirectness	No imprecision	Unlikely	High

Abbreviations: CI: Confidence Interval; EORTC QLQ-C30: European Organisation for Research and Treatment of Cancer Quality of Life Questionnaire Core 30; GRADE: Grading of Recommendations Assessment, Development and Evaluation; IPC: Implantable Port Catheter; MD: Mean Difference; NOS: Newcastle-Ottawa Scale; PICC: Peripherally Inserted Central Catheter; QOL: Quality of Life; RCT: Randomized Controlled Trial; RR: Risk Ratio.

Note: [a] Differences: RR for complications and impact on chemotherapy; MD for cost and EORTC QLQ-C30; HR for complication-free survival.

[b] Risk of bias assessed using the Jadad scale for RCTs.

[c] Publication bias was explored through visual inspection of the funnel plots.

Table S4 EORTC QLQ-C30 and EQ-5D assessment between IPC and PICC regarding the change of quality of life.

Assessment of quality of life	MD [95% CI]	P-value
**EORTC QLQ-C30**		
**Global health status**	3.45 [3.18, 3.72]	< 0.00001
**Functional scales**		
Physical functioning	0.40 [0.17, 0.63]	0.0005
Role functioning	4.36 [3.96, 4.76]	< 0.00001
Emotional functioning	-0.40 [-0.65, -0.15]	0.002
Cognitive functioning	-0.73 [-0.98, -0.48]	< 0.00001
Social functioning	1.69 [1.30, 2.08]	< 0.00001
**Symptoms scales**		
Fatigue	1.16 [0.84, 1.48]	< 0.00001
Nausea and vomiting	2.72 [2.40, 3.04]	< 0.00001
Pain	-1.71 [-2.05, -1.37]	< 0.00001
Dyspnoea	-2.44 [-2.78, -2.10]	< 0.00001
Insomnia	-3.69 [-4.15, -3.23]	< 0.00001
Appetite loss	2.21 [1.76, 2.66]	< 0.00001
Constipation	6.25 [5.84, 6.66]	< 0.00001
Diarrhoea	-3.85 [-4.29, -3.41]	< 0.00001
Financial difficulties	3.91 [3.57, 4.25]	< 0.00001
**EQ-5D**		
Index value	-0.01 [-0.01, -0.01]	< 0.00001
Health state score	2.00 [1.39, 2.61]	< 0.00001

Abbreviations: CI: Confidence Interval; EORTC QLQ-C30: European Organisation for Research and Treatment of Cancer Quality of Life Questionnaire Core 30; EQ-5D: EuroQol 5-Dimension Scale; IPC: Implantable Port Catheter; MD: Mean Difference; PICC: Peripherally Inserted Central Catheter.
